# A Case of Fistula in Ano, with a Few Practical Remarks Regarding the Surgical Operations for Its Relief; as Also the Action of the Sphincter Tertius Described by Hyrtl

**Published:** 1879-03

**Authors:** T. G. Comstock

**Affiliations:** St. Louis, Mo.


*ART II.—A Case of Fistula in Ano, with a few practical remarks regarding the Surgical operations for its relief; as also the action of the Sphincter Tertius described by Hyrtl.* By T. G. COMSTOCK, M. D., St. Louis, Mo.

Cases of fistula in ano, or more correctly fistula in recto, occur so frequently in surgical practice, that I shall take occasion to call the attention of the profession to a few particulars regarding the surgical anatomy of the sphincter muscles.

Some years ago, when a pupil at the University of Vienna, while attending the lectures of Prof. Hyrtl, I first learned the existence of a *Sphincter tertius*. It is remarkable that this sphincter is not mentioned by Gray or other English anatomists. That such a muscle, (whose action is really that of a sphincter), does exist, is to me a matter of no doubt; and if such were not the case, the radical operation for fistula, (dividing the lower sphincters), would be followed by very unpleasant consequences—in other words we should have as a result, involuntary fœcal evacuations. That such an untoward result fortunately does not often occur, every experienced surgeon knows, but the reason for this we shall give by quoting the following, which we have translated from Hyrtl's Anatomy.*

The older surgeons were astonished after having divided the sphincter muscles in operations for fistulæ, that no involuntary discharges of fæces follows. Faget found after removing the lower end of the rectum from a patient, that he could retain his fæces and flatus, and he explained this upon the hypothesis that a new sphincter must have subsequently formed. Houston was not disinclined to believe that the lower portion of the rectum, where a fold occurs as it passes through the pelvic fascia, was surrounded with a development of circular fibres. Lisfranc, who many times extirpated the terminal portion of the rectum, noticed that such patients were not deprived of the power of holding back their stools, and declared it as his opinion, that as a positive necessity a superior sphincter must exist. Likewise every unprejudiced observer must allow of the existence of such a muscle, for the reason that in prolapsus ani, where both the external and internal sphincters are paralyzed, no involuntary stools occur.

"In rupture of the perineum and congenital opening of the rectum into the vagina (cloaca) the same thing happens. Ricord cites the case of a woman, æt. 22, where the rectum opened into the vagina, yet the bowels acted regularly, and what is more remarkable, the husband after having been married three years, had no conception of this abnormal condition in his wife."

"When the index finger is introduced into the rectum of a patient who has had no action from the bowels for a few day, as a rule, just above the anus, no fæces will be found, and yet the column of fæces would naturally sink down to this point, if not held

---

* Handbuch der topographischen Anatomie, Von Josef Hyrtl, Zweiter Band, p. 141, 5te Aufage, Wien 1865.

back by an opposing circular muscle. Kohlrausch opposed this view, which presupposes the existence of a third sphincter, because he found upon dead subjects, as well as in patients, hard scybala in the lower portion of the rectum; but I take occasion to mention that the existence of fæces in the rectum upon subjects, simply proves that the sphincter tertius no longer acts, and the same thing in the living, (in patients), may be the result of diseased conditions, and which affords an example of an exception to the rule. Enemata which are not introduced high enough into the rectum, are liable to come away immediately; on the contrary, if the canule (extremity) of the syringe is pushed up sufficiently high the injection will be retained a longer time. Dr. O'Beirn called attention to the fact that an elastic tube can be introduced quite a distance into the rectum, before any flatus is given off, and then the discharge comes suddenly. All these observations make it probable, *a priori* that at a certain distance above the internal sphinter ani, a third sphincter must exist. Nelaton, and Velpeau have demonstrated the existence of it, as a thickened band of muscular fibres, four inches above the anus. This muscular development, is not always easy to find. To find it upon the cadaver, care should be taken that the rectum is not forcibly distended with air.

In order to demonstrate it well, the rectum should be cut upwards longitudinally, and stretched upon a board, and the several layers carefully dissected off, until the muscular layer is reached, when the sphincter tertius, if present, will be seen as a broad bundle of thickly conglomerated muscular fibres. Not unfrequently this investigation will be fruitless of a result, but the physiological fact that there are developed muscular fibres encircling the rectum at this point, is not to be doubted. In one instance I have publicly demonstrated the existence of the fibres of the sphincter tertius taking their origin from the sacrum.

This third sphincter does not permit the excrements, (fæces), which are in the sigmoid flexure and are pressing down, to reach the lower rectum. Only when the desire for an evacuation exists, does it relax and allow the fœcal column to come down on to the lower sphincters. These latter can voluntarily keep back the stools for a long time, and are assisted in their efforts by the levator ani muscle, as likewise by the buttocks, (nates), firmly pressed together, so that when one is unfortunately in such a critical situation, (for obvious reasons) he takes care not to take long steps, or to run. At last these muscles from having such an unusual strain upon them, become paralyzed, and then follows, what, under such circumstances is of course unavoidable. When the lower end of the rectum is removed, or the sphincters are divided as in the operation of "rectal fistula, then the patient will not be afflicted with the most hopeless and disgusting of all ailments, viz., involuntary stools; for when the slightest desire for a stool is experienced, and the upper sphincter is relaxed, the evacuation below is being accomplished, because simultaneously the two lower sphincters will involuntarily be relaxed."

I should perhaps mention that Dr. James R. Chadwick, of Boston, in a very elaborate article,* regards the sphincter tertius, as "a collection of constricting bands, and a part of the general circular layer of muscles, whose function is to *dilate before*, and *contract behind* the scybala, thereby propelling them on their way, and not retarding them." He proposes to call this sphincter, a "*Detrusor faecium.*" I did not intend to discuss Dr. Chadwick's essay, but the facts adduced by Hyrtl are undoubtedly true. I was called in consultation in May last, to see a gentleman, who to avoid a collision, jumped from a railroad train going at the rate of forty-five miles an hour; he struck with great violence upon the end of a railroad iron, which seems to have raised up, and which penetrated him in the region of the perineum. The injury was so great, that it seemed almost as if he was cleft in twain; suffice it to say, the lower end of the rectum was so contused and injured, that it sloughed away. Fortunately the patient had a short time previous to the accident, passed a large stool, and notwithstanding the severity of the shock and loss of blood, with the subsequent surgical fever, he had no operation from his bowels for some seven days. This gentleman has recovered, but the lower portion of the rectum is quite gone, and yet he can control his stool. Is not this an instance of the existence of Hyrtl's sphinc-

* Transactions of the American Gynecological Society, Vol. 2, p. 43. Boston, 1878.

ter? When we have a fistula of the rectum, why is it necessary to divide the sphincter in order to enable the parts to heal.

1st. Because all efforts at healing as a general rule fail, unless we can expose the pyogenic membrane which often lines the fistulous tract. 2d. The healing process is prevented by the constant motions of the sphincter and levator ani muscles; because with every act of respiration they contract, and thus prevent healing; and to do away with this effectually we must make a section of the sphincter. Occasionally cases are reported where a cure results without dividing the sphincter.

Sir Astley Cooper mentions two cases; Ashton* mentions several in his large experience; Dr. Ordway, of Boston,† reports that he has cured many cases by injection with sesqui-carbonate of potash (vegetable caustic); however, such cures are, in the experience of the profession, exceedingly exceptional. In my own experience I know of only one case thus cured:—it was a clergyman, who refused to be "cut," and after one year reported to me that he was cured by injections and pressure combined; the pressure was by means of a compressed sponge introduced from time to time within the anus. Patients fear the knife, and willingly resort to salves for relief, and in this respect history repeats itself for the past two hundred years. Louis XIV., King of France, was so unfortunate as to be afflicted with a fistula. His medical attendant seems to have been a real practical surgeon, versed well in surgical pathology, as well as therapeutics. He diagnosticated the ailment of the King, and informed His Royal Highness that the cure could be accomplished only through a surgical operation. The King was very shy of being cut, and as various methods of treatment had been proposed for him, "without any resort to the knife," he was shrewd enough to object to have them tried upon his own Royal person, until he should have seen their good effects upon others; and he accordingly ordered a number of his subjects suffering from fistula to be treated in accordance with the different plans which had been suggested. Among other cures, the mineral springs of Barége, as also the waters of Bourbon were proposed, and to these

---

* Fistula in Ano, and Hemorrhoidal Affections, London, 1873.
† *Boston Medical and Surgical Journal*, vol. 99, p. 657.

springs he sent the patients accompanied by a physician, whose province it was to observe the results of the drinking of, and the bathing in the waters, as well as the injecting of the same waters into the fistulæ. After some months, these invalids were all brought back to Paris, and the fistulæ were nearly as bad as when they went thither. Next, chambers or wards were fitted up at royal expense, and the patients with fistulæ were there carefully treated in accordance with the various methods of cure of pretenders, who recommended ointments, salves and solutions for injecting, as likewise internal injections. A whole year was spent in this way in experimenting, but not one of the patients was cured by any of these means. At last the king gave in to his surgeon, Mons. Felix, who operated upon him Nov. 21st, 1687, making the identical operation of the present day—freely opening the sinus into the gut, and cutting through the sphincter. The operation was a success, and the king was, in a short time, perfectly cured. I have taken the liberty of calling attention to the above case, which may be of historical interest to the surgeon of the present day, and may be regarded as classical.

Fistulæ of the rectum may occur in the young or old, and may accidently happen to those leading a pure and regular life; but high livers and those who are intemperate are especially liable to them.

The following case came accidently under the attention of the writer: In August last, while on a visit at Le Roy, N. Y., I was consulted by Miss ———, a young lady aet. 26.

She informed me that some 18 months previously, from the effects of a fall, she had suffered from an abscess in the ischio-rectal region, which had finally terminated in a double fistula. For this affection she had been to a "Cure" for five months, and was there treated by the lady physician in charge, who had improved her general health very much, and had endeavored to heal the fistulæ by various injections, and other applications, but without effect. Upon examining the case, I found two fistulous openings upon each side of the posterior commissure of the vagina, extending into the rectum. I introduced a probe into one opening, and found a fistulous tract terminating in the rectum, at a distance of over two and one-half inches above the anus. I then introduced a second probe into the opposite opening, and succeeded in passing it through the tract of the same opening also into the rectum; with one index finger in the rectum, I made the end of each probe impinge upon it. Here then was a double fistula with one common opening in the rectum. After this diagnosis, I announced to the young lady's mother that her daughter's ailment could be easily relieved by a surgical operation, the nature of which I explained to her.

Several objections were made to the operation, and I was solicited to try and cure it by other means.

The first objection was, that such an operation was not approved of by her last medical advisor, who proposed to cure the fistulæ by placing the patient under the influence of ether, and then forcibly distending the sphincter, to paralyze the same, and afterwards to treat the fistulous tracts by injections, and thereby hoped ultimately to affect a cure.

2d. Her last medical advisor regarded the patient as "a bad subject for the healing process, should any surgical cutting be done."

3d. The patient herself objected to the knife. As I had firmly stated that no cure could follow any procedure whatever, short of a radical operation, and as the patient was a near relative of mine, and therefore feelings of delicacy were involved in the matter, I proposed that the young lady and her mother should accompany me to Buffalo, to consult Dr. J. F. Miner.

They accordingly did this, and Dr. Miner was consulted Sept. 23d, and he quite agreed with me in the diagnosis, and approved of the treatment as above proposed;—in other words Prof. M. said, "it was a case to be treated in accordance with the principles and practices of surgery," that the pyogenic surface of the fistulous tracts should be freely and completely exposed, and incision into the common opening in the rectum, and the sphincter divided. As the patient had at all hazards objected to the knife, as a *substitute*, I suggested to Dr. Miner the feasibility of operating by ligature, to which he assented. The principle of this operation by ligature is as old as Hippocrates, who used the *seton* in fistula. For improvements in the use of the ligature, we are indebted to

Dr. Dittel, of Vienna, who first proposed the *elastic ligature*, which is made of india-rubber, the size of a whip-cord. The end of the ligature is split with a pair of scissors; then it is threaded in the eye of a good sized silver probe; then the probe, armed with the ligature, is introduced into the fistula and pushed into the opening in the rectum, and brought out through the anus; then the two ends are passed through a little leaden ring (not unlike to a good sized buck-shot with a hole through it), and stretched to its maximum tension, then the ring is crushed or clamped with strong forceps or pincers in such a wise, that the fistula is included or strangulated within an elastic noose, and this tension steadily maintained until the ligature in time performs the part of a knife by cutting through the sphincter, when it is discharged. This new method by the elastic ligature has not only the sanction of Dittel the inventor, but of Allingham and Sir Henry Thompson.*

Having given my reasons for using this ligature, supported by surgical authorities, I accordingly made trial of it in this case.

I returned to Le Roy with the patient, and, assisted by Dr. R. Williams, proceeded to make the operation, Sept. 26th, 1878. The bowels were evacuated early in the day with an enema, and Dr. Williams administered to her, by inhalation, a mixture of three parts of ether to one of chloroform; she soon came under its influence, when I introduced an elastic ligature into each sinus, and passed them through the common opening into the gut; the ends of each were then brought through the ring of lead and each one separately clamped, as I have above described. The patient, although delicate and nervous, had no untoward symptoms after the operation, with the exception of a diarrhœa on the fourth day, which soon subsided. One ligature cut through on the eighth day, and the other ligature on the tenth day. The patient was quite comfortable through the whole time of treatment, excepting the slight looseness of the bowels above mentioned, and made a rapid recovery. A little gap or cleft made by the division of the sphincter did not entirely heal for some weeks, but she always had perfect control of her bowels, and at this time, three months after the

* See Braithwaite's Retrospect, Part 69, 1875, page 108 and 179. Also Elastic Ligature in anal fistula. Directions for its Use, by Allingford. *Phila. Med. and Surg. Reporter*, vol. 33, p. 153 and 110.

operation, she is quite well. After the operation I was obliged to return to St. Louis, but left the patient in charge of Dr. R. Williams, a resident practitioner in Le Roy for twenty-five years past, and to whose careful attention the favorable issue of the case is not a little due.

I am quite certain that experienced surgeons will not give up the knife for the elastic ligature, and the writer of this does not wish to be considered as recommending it above the knife; but it certainly has its advantages, and these I shall take the liberty to enumerate.

SUMMARY OF THE ADVANTAGES OF THE ELASTIC LIGATURE.

1. Applicable as a substitute for the knife when patients are delicate, timid, possibly phthisical, and positively decline "to be cut."

2. Appropriate when the opening in the gut is situated unusually high up.

3. Operation followed by no hemorrhage.

4. Patients not necessarily confined to bed after the operation, but may go in the air, and in some instances, even pursue their ordinary avocations.

6. Little suppuration after the operation.

7. Recovery usually rapid.

8. Operation in many cases may be performed at the surgeon's office, and patient get up from the operating chair and go home without discomfort.

Lastly, Dr. Wm. Allingham says: "I do not consider the elastic ligature can ever supplant the knife in the treatment of fistulous sinuses. In complicated cases the knife must be depended upon mainly, but I am of opinion that the india-rubber ligature is valuable in many cases as a substitute, and in others as an auxiliary, to the usually employed method of excision."

